# The Role of Trastuzumab in Patients with HER2 Positive Small (pT1mi/a) Breast Cancers, a Multicenter Retrospective Study

**DOI:** 10.3390/cancers13225836

**Published:** 2021-11-21

**Authors:** Andrea Villasco, Silvia Actis, Valentina Elisabetta Bounous, Fulvio Borella, Marta D’Alonzo, Riccardo Ponzone, Corrado De Sanctis, Chiara Benedetto, Nicoletta Biglia

**Affiliations:** 1 Academic Division of Obstetrics and Gynaecology-A.O. Ordine Mauriziano, University of Turin, 10128 Turin, Italy; andrea.villasco@unito.it (A.V.); silvia.actis@unito.it (S.A.); valentinaelisabetta.bounous@unito.it (V.E.B.); marta.dalonzo@unito.it (M.D.); 2 Gynaecology and Obstetrics 1-City of Health and Science, University of Turin, 10126 Turin, Italy; fulvio.borella@unito.it (F.B.); chiara.benedetto@unito.it (C.B.); 3 Gynecological Oncology, Candiolo Cancer Institute, FPO-IRCCS, 10060 Candiolo, Italy; riccardo.ponzone@ircc.it; 4 Breast Unit, Department of Gynecology and Obstetrics, City of Health and Science, University of Turin, 10126 Turin, Italy; corrado.desanctis@unito.it

**Keywords:** trastuzumab, HER2 positive, pT1mic-pT1a, breast cancer

## Abstract

**Simple Summary:**

Treatment of small HER2+ breast cancers with adjuvant Trastuzumab is still controversial. This study aims to measure the effect of Trastuzumab in early stages of breast cancer (pT1mic/a pN0/1mi) in terms of disease recurrence and to identify which factors most affect the prognosis of small HER2+ tumors. We retrospectively selected and reviewed 100 HER2+ pT1mic-pT1a breast cancer patients with a median follow-up of 86 months. In our study the primary outcome was the disease recurrence rate, which appeared to be significantly lower among patients who received adjuvant Trastuzumab. Among the patients who did not receive adjuvant Trastuzumab, HR− HER2+ tumors showed a risk seven times higher of relapse. The results of our study indicate that adjuvant Trastuzumab reduces the risk of developing a disease recurrence even in small HER2+ tumors. Adjuvant targeted therapy should be considered in patients with HR− HER2+ tumors, the category with the highest risk of recurrence.

**Abstract:**

The treatment with adjuvant Trastuzumab in patients diagnosed with HER2+ small breast cancers is controversial: limited prospective data from randomized trials is available. This study aims to measure the effect of Trastuzumab in the early stages of breast cancer (pT1mic/a pN0/1mi) in terms of disease recurrence and to identify which are the factors that most affect the prognosis of small HER2+ tumors. One hundred HER2+ pT1mic-pT1a breast cancer patients who were treated in three Turin Breast Units between January 1998 and December 2018 were retrospectively selected and reviewed. Trastuzumab was administered to 23 patients. Clinicopathological features and disease-free survival (DFS) were compared between different subgroups. The primary outcome was the disease recurrence rate. Median follow-up time was 86 months. Compared to pT1a tumors, pT1mic lesions had a higher tumor grade (84% of pT1mic vs. 55% of pT1a; *p* = 0.003), a higher Ki-67 index (81% vs. 46%; *p* = 0.007) and were more frequently hormone receptor (HR) negative (69% vs. 36%, *p* = 0.001). Disease recurrence rate was significantly lower among patients who received adjuvant Trastuzumab (*p* = 0.02), with this therapy conferring an 85% reduction in the risk of relapse (HR 0.15; *p* = 0.02). Among the patients who did not receive adjuvant Trastuzumab, the only factor significantly associated with an increased risk of developing a recurrence was the immunohistochemical (IHC) subtype: in fact, HR− HER2+ tumors showed a risk seven times higher of relapsing (HR 7.29; *p* = 0.003). Adjuvant Trastuzumab appears to reduce the risk of disease recurrence even in small HER2+ tumors. The adjuvant targeted therapy should be considered in patients with HR− HER2+ tumors since they have the highest risk of recurrence, independently from size and grade.

## 1. Introduction

The implementation of mammographic screening during the last decades has led to changes in the size-specific distribution of breast cancer, with increased detection of small breast tumors. Nowadays, carcinomas up to 1 cm in size represent 40% of all screening diagnosed breast cancers [1].

The American Joint Committee on Cancer Staging Manual lists “T1mic” in the TNM classification and defines microinvasion as the extension of cancer cells beyond the basement membrane into the adjacent tissue with focus up to 1 mm in the greatest dimension. Invasive components exceeding this size are classified as T1a if the diameter ranges from 2 to 5 mm [2].

Microinvasive carcinomas are rare, and the survival outcomes are controversial, often conformed to those of ductal carcinoma in situ (DCIS) [3].

Certain subgroups of patients with small breast cancers have a greater risk of recurrence. Factors found to be associated with poorer outcomes are high tumor grade, hormone receptors (HR) negative status, young age at diagnosis, high Ki-67 values, lympho-vascular invasion, and human epidermal growth factor receptor 2 (HER2) expression [4,5,6].

The HER2/neu is a significant predictor of overall survival (OS) and time to relapse in patients with breast cancer [7]. Since the early 2000s, a recombinant humanized monoclonal antibody (Trastuzumab) designed to selectively target the extracellular HER2 receptor has been available to treat patients with HER2-positive tumors [8]. Trastuzumab therapy is widely considered a highly effective treatment with a favorable benefit/risk profile, leading to significant gains in OS and disease-free survival (DFS) [9,10].

In tumors <1 cm without lymph node involvement (pT1a/b N0) HER2 overexpression and/or gene amplification occurs in 6–10% of cases [11]. Several reviews of the literature [11,12,13] and retrospective studies [14,15] have shown that HER2+ tumors smaller than 1 cm have a worse prognosis than their HER2− counterparts, showing increased risk of relapse and decreased survival. Although HER2+ status in small breast neoplasms is associated with a worse prognosis, there is currently limited prospective data from randomized trials regarding the benefit of adjuvant Trastuzumab in small, node-negative, breast tumors. In fact, in a recently published metanalysis, Trastuzumab was shown to significantly reduce the risk of recurrence also in very small HER2+ breast cancer patients (HR 0.61, 95%CI 0.38–0.99). However, this result is derived from a subgroup analysis conducted on 1095 merged pT1a and pT1b patients, with a limited number of events [16]. According to the NCCN Guidelines 2021, the decision to prescribe a Trastuzumab-based therapy in this group of patients must balance the known toxicities of this treatment and the uncertain benefits that can be achieved [17].

Current data suggest that negative prognostic factors, such as high nuclear grade, high proliferative index, younger age, and HR negativity, should be considered in treatment decisions regarding small HER2+ tumors [18,19]. 

The present study aims at measuring the effect of Trastuzumab in very small early breast cancers (pT1mic/a pN0/1mi) in terms of disease recurrence, and at identifying the factors that most affect the prognosis of small HER2+ tumors.

## 2. Materials and Methods

### 2.1. Patients

The study was conducted retrospectively on 100 consecutive HER2+ breast cancer patients with available follow-up, and who underwent surgery between January 1998 and December 2018 in three Turin Breast Units (“A.O. Ordine Mauriziano”, “Candiolo Cancer Institute-IRCCS” and “Città della Salute”). 

Tumors eligible for this study were micro-invasive cancers 0.1 cm or less in dimension (pT1mic) or up to 0.5 cm in dimension (pT1a) at the pathologic examination. Regarding lymph node involvement, we included node-negative tumors (pN0), isolated tumor cells (pN0i+), and lymph node micrometastases up to 0.2 cm in size (pN1mi).

According to the American Society of Clinical Oncology (ASCO), HER2-positivity was defined as either the overexpression of HER2 protein, “+++” assessed by immunohistochemistry, or “++” with ascertained amplification of HER2/neu gene at fluorescence in situ hybridization (FISH) assay [20]. Only HER2-positive cancers were included in this study.

Exclusion criteria were: bilateral breast cancer, use of neoadjuvant therapy, and lymph node metastasis above 0.2 cm in size (pN1a upwards).

Two treatment groups were defined according to the administration of target therapy with the anti-HER2 antibody Trastuzumab. Treatment decision on adjuvant Trastuzumab was routinely taken by a multidisciplinary team. Genomic testing was not available in the Breast Units at the time considered. Patients underwent clinical follow-up examination every 6 months in the first 5 years after surgery and every year subsequently. Annual mammography was indicated for all women; further examinations (liver ultrasonography, chest X-ray and bone scan) were performed only in case of clinical suspicion.

### 2.2. Statistical Analyses

Statistical analyses were conducted using SPSS Statistics 20.0 (IBM, New York, NY, USA) and STATA 16.0 (StataCorp LLC, College Station, TX, USA) software.

The Kolmogorov–Smirnov test was used to assess the distribution of the quantitative variables under study.

Pearson’s chi-squared test (χ^2^) was used for discrete variables to study significant differences between pT1mic vs. pT1a populations and Trastuzumab vs. non Trastuzumab populations, while Student’s t-test was used for continuous variables. 

The primary outcome was the disease recurrence rate. Recurrence-free survival (RFS), defined as the time elapsed from surgery to the development of local or distant relapse of the disease, was assessed using the Kaplan–Meier curve analysis with the log-rank test.

The Cox proportional hazards model was used to estimate the hazard ratio (HR) and confidence intervals (CI). The Fine-Gray competing risk analysis was performed to provide a better estimation for the effect of Trastuzumab on the main outcome in the presence of competing risk events.

## 3. Results

### 3.1. Sample Description

Among the 100 enrolled patients, the median age at surgery was 57 years. Fifty-eight patients had pT1mic tumors, and 42 patients had pT1a tumors. As for immunohistochemical (IHC) subtype: 45 tumors were HR+ HER2+, while 55 tumors were HR− HER2+. Half of the patients underwent mastectomy, since often early breast tumors were identified in the context of an extensive in situ neoplasia. Details are shown in Table 1.

### 3.2. Tumor Characteristics: pT1mi vs. pT1a

Compared to pT1a tumors, pT1mic lesions had a higher tumor grade (84% of pT1mic vs. 55% of pT1a, *p* = 0.003), and more often showed a high Ki-67 index (81% vs. 46%; *p* = 0.007). A significantly higher percentage of pT1mic neoplasms was HR− HER2+ when compared to pT1a breast cancers (69% vs. 36%, *p* = 0.001). The details are shown in Table 2.

### 3.3. Trastuzumab Administration and Disease Recurrence Analysis

Trastuzumab was administered to 23 patients: 10 pT1mic and 13 pT1a patients. Chemotherapy schemes associated with trastuzumab administration are detailed in Table 3. At multivariate logistic regression, Trastuzumab prescription did not appear to be related to patient’s age, size, grade, IHC-subtype, type of surgery, ki67 value, nodal status, use of radiotherapy, or endocrine therapy, as shown in Table 4.

**Figure 1:**
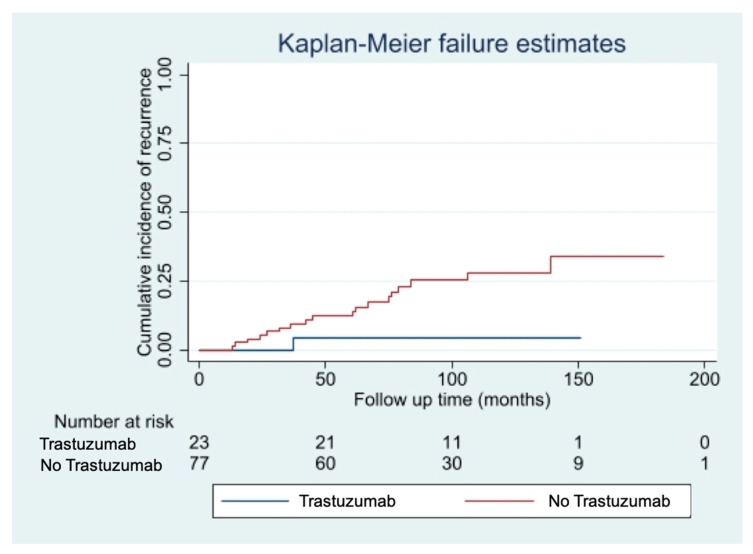
Cumulative incidence of disease recurrence according to treatment with Trastuzumab.

The median follow-up time was 85 months [10–213 months]. We observed 20 cases of disease recurrence (16 loco regional recurrences, 4 distant metastases). Fourteen cases of disease recurrence (24%) occurred in the pT1mic subgroup, while 6 cases (14%) occurred in the pT1a subgroup. Eight deaths were recorded during follow-up: three patients died of breast cancer, five of intercurrent events, all belonging to the non-trastuzumab group. Among patients with small (pT1mic/a pN0/1mi) HER2+ breast cancers, disease recurrence rate was significantly lower among patients who received adjuvant Trastuzumab (4% Trastuzumab group vs. 26% non-Trastuzumab group, *p* = 0.02), with this therapy conferring an 85% reduction in the risk of relapse (HR 0.15; 95%CI 0.10–0.97; *p*-value: 0.048). The curves of cumulative incidence of recurrence are shown in Figure 1.

**Figure 2:**
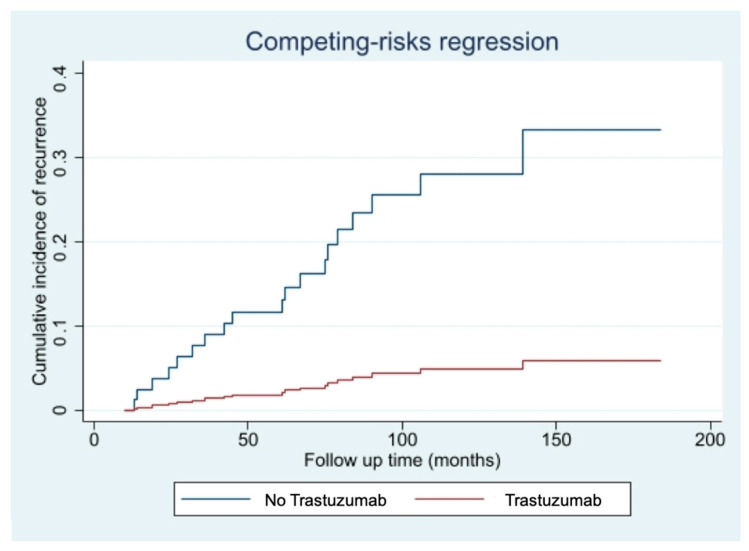
Cumulative incidence of disease recurrence according to treatment with Trastuzumab-Fine-Gray competing risk analysis for intercurrent non-breast cancer-related deaths.

The competing risk events analysis, performed to account for the possible effect of the 5 non breast cancer related-deaths, confirmed the protective effect offered by the Trastuzumab treatment (HR 0.14; 95%CI 0.02–0.90; *p*-value: 0.039), as shown in Figure 2.

### 3.4. Disease Recurrence Risk Factors

**Figure 3:**
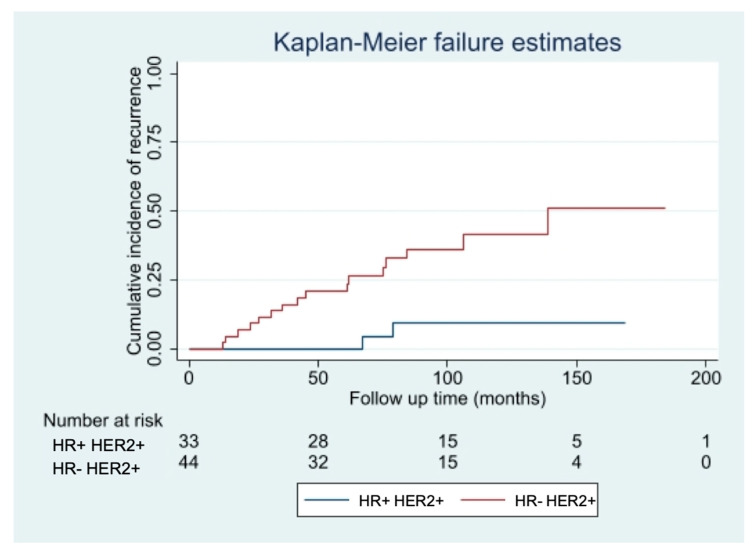
Risk of relapse in patients not treated with Trastuzumab with HR− vs. HR+ tumors.

Among the patients who did not receive adjuvant Trastuzumab, the only factor significantly associated with an increased risk of developing a recurrence was the IHC-subtype, with hormone receptor negative (HR− HER2+ tumors having a risk seven times higher of relapsing (HR 7.29, 95% CI 1.68–31.60; *p* = 0.003), independently from patient’s age, pT, pN, Ki67 value, type of surgery and adjuvant treatments. The curves of cumulative incidence of recurrence are shown in Figure 3.

## 4. Discussion

Studies conducted to define the optimal treatment of microinvasive and small breast cancers have been greatly limited by the generally good outcome of these cancers; therefore, although the prognosis of pT1mic/pT1a is considered favorable, their clinical management and behavior are less clear.

pT1a tumors account for about 3% of all diagnosed breast tumors [21]. Patients with pT1a breast cancers show an excellent prognosis, with 5-years breast cancer-specific survival rates ranging from 87% to 99% [22,23]. pT1mic breast cancers are an uncommon pathologic entity, accounting from 0.7% to 3.4% of all breast cancer cases. They are predominantly identified in the setting of extensive DCIS [4,5,6]. A SEER database analysis published in 2017 including 8863 pT1mic breast cancers showed that microinvasive breast cancers have worse breast cancer-specific survival and OS compared to DCIS. The breast cancer-specific mortality rate for pT1mic was nevertheless low (4.08%) [24], similar to the 4% rate observed for 51,246 pT1a-b patients in another SEER database analysis published in 2007 [6]. It is interesting to notice that studies have observed that the overall incidence of HER2 positivity in pT1mi tumors ranges from 37% to 57.4% [25,26], much higher than in larger size invasive carcinoma (10–20%) and pure DCIS (13–20%) [27,28].

In this study, we have focused specifically on smaller tumors within the category of early breast cancers, including exclusively HER2-positive pT1a and pT1mic tumors. We have observed that pT1mic tumors were mostly HR− HER2+ lesions. Literature regarding this aspect is limited and controversial. Three different retrospective studies found that among the HER2+ pT1mic tumors, the majority were HR−, differently from pTis and pT1a-b that were more frequently Luminal tumors [26,29,30]. On the contrary, another retrospective study found no significant differences in HR−positivity rates in HER2+ pT1mic cancers when compared with pTis and pT1a tumors [31].

In addition to being frequently HR−, we have observed that pT1mic patients had more frequent high-grade tumors with a higher proliferative index compared to pT1a tumors. Similar data were observed by Costarelli et al. on a retrospective analysis from 10 breast cancer centers on 17,431 pTis, pT1mic, and pT1a patients, in which the pT1mic patients were the ones with the most unfavorable biological features [28].

In a systematic review including 7 studies about the role of HER2/neu as a prognostic factor for survival and relapse in pT1a-bN0M0 breast cancers, HER2 positivity in small breast tumors appeared to be associated with a worse outcome regarding breast cancer-specific survival, relapse-free survival, and distant relapse-free survival [13]. In the early 2000s, a recombinant humanized monoclonal antibody (Trastuzumab) designed to selectively target the extracellular HER2 receptor was made available to treat patients with HER2+ tumors [8]. Several large trials have evaluated the association of Trastuzumab into adjuvant chemotherapy regimens, demonstrating a marked improvement in both DFS and OS in patients who had HER2+ diseases. Data from these studies were summarized in a meta-analysis, which showed that patients who had HER2+ breast cancer and were treated with Trastuzumab had approximately a 50% reduction in the risk of early recurrence and mortality, irrespective of nodal status [10]. However, most of these trials consistently excluded patients with tumors that were 1 cm or smaller (pT1mic, pT1a, pT1b), even if different reviews of the literature [11,12,13] and retrospective studies [14,15] evaluating the natural history of small breast cancer based on biological characteristics, showed that HER2+ tumors smaller than 1cm have a worse prognosis than their HER2- counterparts. 

According to the NCCN Clinical Practice Guidelines in Oncology Recommendation for Adjuvant HER2-Targeted Therapy, Trastuzumab and chemotherapy should be used for women with HER2-positive tumors smaller than 0.6 cm with positive lymph nodes [17]. Beyond this recommendation, indications on whether to give Trastuzumab in patients with small node negative HER2+ breast cancers are not homogeneous in the literature. The choice to administer such treatment is often made considering other clinicopathological characteristics. In a meta-analysis carried out by Lee et al. [29] on seven controlled studies on a total of 1181 patients pT1a-b pN0 HER2+, patients in the Trastuzumab arm had poorer pathological profiles, such as grade 3, and HR− tumors. In our study, on the contrary, patients who were given Trastuzumab and patients who did not receive it did not show significant differences in size, grade, IHC-based subtype, mean age at diagnosis, type of surgery, use of radiotherapy, and hormone therapy. These results reflect the disarray observed in daily clinical practice concerning the prescription of target therapy for small HER2+ tumors, due to the little and contradictory data available. The same metanalysis [29] showed that adjuvant treatment with Trastuzumab was associated with a significantly reduced risk of overall recurrence in pT1a-b N0M0 HER2+ patients. Similar results were obtained by Zhou et al., in a metanalysis of eight studies, in which Trastuzumab led to a significant improvement in DFS. Moreover, Zhou et al. performed an exploratory metanalysis by integrating the BCIRG006 trial [32], a randomized clinical trial of adjuvant Trastuzumab which recruited HER2+ pT1a-b patients irrespective of nodal status, which revealed a marked improvement in DFS with the addition of Trastuzumab. From this evidence, it follows that the efficacy of adjuvant Trastuzumab might not vary widely by nodal status in pT1a-b breast cancer patients [33].

O’Sullivan and colleagues conducted a meta-analysis on five randomized trials to compare the efficacy of adjuvant therapy with or without Trastuzumab in patients with HER2+ breast cancer with tumor size ≤2 cm; they showed that the patients derived a substantial DFS and OS benefit from adjuvant Trastuzumab. Interestingly, the proportional benefit offered by adjuvant Trastuzumab seemed to be the same regardless of tumor size or nodal status [34].

More recently, the EBCTG published a metanalysis on individual patient’s data from 7 randomized trials. A subgroup analysis included 1095 patients with tumor size ranging from 1 to 10 mm, and Trastuzumab was shown to significantly reduce the risk of recurrence even in small HER2+ breast cancer patients (HR 0.61, 95%CI 0.38–0.99). This is so far the only good quality prospective data available on Trastuzumab treatment in very small breast tumors [16].

In agreement with the evidence presented in current literature, in our study disease recurrence rate was significantly lower among pT1mic-pT1a patients who received adjuvant Trastuzumab, independently from nodal status, with an 85% reduction in the risk of relapse. 

These results are consistent with the emerging evidence that it is not tumor size alone but its biology that better delineates the prognosis of small tumors [21]. Joerger et al., in their review of the literature, suggested that tumor biology is a more relevant predictor for the risk assessment in small HER2+ tumors than tumor size. They advocated that adjuvant treatment might also be considered in patients with HER2+ tumors <6 mm when factors such as HR negativity, increased Ki-67 and/or poor nuclear grade suggest aggressive tumor biology. Our study partially corroborates this input. In fact, among patients who did not receive adjuvant Trastuzumab, IHC-subtype was significantly associated with an increased risk of developing a recurrence, with HR− HER2+ tumors having a risk seven times higher of relapse.

Opposite results were obtained in a retrospective analysis by Tongela et al. [35] on 128 women with stage I HER2+ breast cancers, in which there were no significant differences in relapse free survival and OS for the women with tumors <1 cm treated with Trastuzumab. Analogous results were reported by other authors in a multi-institutional retrospective study [36].

The use of target therapy in small HER2+ early breast cancers remains controversial. Adjuvant Trastuzumab has been shown to reduce the risk of developing a disease recurrence even in small, pT1a-b, and in our study pT1mic, HER2+ tumors, with a beneficial effect irrespective of nodal status and size. 

### Limitations

The major limitationa of this study are the pure retrospective design and the limited number of events recorded. Data on chemotherapy and Trastuzumab side effects were not available, thus preventing us from eliciting their incidence and evaluating the risk/benefit ratio.

## 5. Conclusions

There is more and more evidence in the literature that it is not tumor size alone but the biology of small tumors that influences their prognosis. In agreement with the already available data, we have shown that HR− HER2+ pT1mi and pT1a tumors have a significantly higher risk of recurrence. There are early prospective data that Trastuzumab is effective in preventing disease recurrence also in very small HER2+ breast cancer. Our retrospective study supports this evidence, but further randomized prospective clinical trials including larger sample size are needed to clarify the benefit of Trastuzumab in small HER2+ early breast cancers. 

## Figures and Tables

**Table 1:** Data of the study population (total of 100 patients).

Median Age at Diagnosis (Years)	57	SD 13
	*n* = 100	%
Menopausal status at diagnosis		
Pre-menopausal	34	34
Post-menopausal	66	66
pT status		
pT1mic	58	58
pT1a	42	42
Nodal status		
pN0	92	92
pN1mi	8	8
Grading		
G1	0	0
G2	24	24
G3	72	72
Unknown	4	4
Ki-67		
Low < 20%	20	20
High > 20%	35	35
Unknown	45	45
IHC subtype		
HR+ HER2+	45	45
HR− HER2+	55	55
Type of surgery		
Conservative surgery	51	51
Mastectomy	49	49
Radiation Therapy		
Yes	46	46
No	54	54
Hormone therapy		
None	65	65
Aromatase Inhibitors	16	16
Tamoxifen	19	19
Chemotherapy		
Yes	27	27
No	73	73
Trastuzumab therapy		
Yes	23	23
No	77	77
Relapse		
Local recurrence	16	16
Distant metastases	4	4
No	80	80

Sd: standard deviation; pT1mic: lesions with invasive focus up to 1 mm in size; pT1a breast tumors with invasive components from 2 to 5 mm; pN0 absence of lymph node involvement; pN1mi: lymph node micrometastases (maximum size between 0.2 mm and 2 mm); IHC-subtype immunostochemical subtype; HR: hormone receptor.

**Table 2:** Comparison between pT1mic and pT1a subgroups.

Categories	pT1mic		pT1a		
	*n* = 58	%	*n* = 42	%	*p*-Value
Menopausal status					0.508
Pre-menopausal Post-menopausal	16 41	28 71	16 25	38 60	
Grade					0.003
1	0	0	0	0	
2	8	14	16	38	
3	49	84	23	55	
Ki67					0.007
Low < 20%	5	9	15	54	
High > 20%	22	81	13	46	
IHC- subtype					0.001
HR− HER2+	40	69	15	36	
HR+ HER2+	18	31	27	64	
Trastuzumab therapy Yes No	10 48	17 83	13 29	31 69	0.149

HR: Hormone Receptors; IHC-subtype: immunohistochemical subtype.

**Table 3:** Chemotherapy schemes associated with trastuzumab administration.

Categories	pT1mic		pT1a		
	*n* = 10	%	*n* = 13	%	*p*-Value
Chemotherapy schemes					0.85
4 cycles of Docetaxel 75 mg/m^2^ + Cyclophosphamide 600 mg/m^2^ and Trastuzumab for 1 year	9	90	12	92	
Paclitaxel 80 mg/m^2^ weekly for 12 weeks and Trastuzumab for 1 year	1	10	1	8	

**Table 4:** Clinic-pathological features of patients who received Trastuzumab vs. patients who did not receive Trastuzumab.

Categories	Trastuzumab Group		Non-Trastuzumab Group		
	*n* = 23	%	*n* = 77	%	*p*-Value
Menopausal status at diagnosis					0.71
Pre-menopausal	7	30	25	32	
Post-menopausal	16	70	50	65	
Unknown	0	0	2	3	
Size					0.11
pT1mic	10	43	48	62	
pT1a	13	57	29	38	
Lymph node status					0.38
pN0	20	87	72	94	
pN1mi	3	13	5	6	
Grade					0.07
G1	0	0	0	0	
G2	9	39	15	20	
G3	14	61	58	75	
Unknown	0	0	4	5	
Ki 67					0.25
Low < 20%	3	14	17	22	
High > 20%	10	43	25	32	
Unknown	10	43	35	46	
IHC- subtype					0.43
HR+ HER2+	12	52,2	33	43	
HR− HER2+	11	47,8	44	57	
Type of surgery					0.73
Conservative surgery	11	48	40	52	
Mastectomy	12	52	37	48	
Radiation Therapy					0.78
Yes	10	43	36	47	
No	13	57	41	53	
Hormone therapy					0.19
Yes	11	48	25	32	
No	12	52	52	68	

pT1mic: lesions with invasive focus up to 1 mm in size; pT1a breast tumors with invasive components from 2 to 5 mm; HR hormone receptors; IHC-subtype: immunostochemical subtype; RT: radiation therapy; CT chemotherapy; pN0 absence of Lymph node involvement of the tumor; pN1mi Lymph node micrometastases (maximum size between 0.2 mm and 2 mm).

## Data Availability

The datasets generated during and/or analysed during the current study are available from the corresponding author on reasonable request.
